# The cost-utility of an intervention for children and adolescents with a parent having a mental illness in the framework of the German health and social care system: a health economic evaluation of a randomized controlled trial

**DOI:** 10.1186/s13034-023-00693-w

**Published:** 2023-12-21

**Authors:** Tamara Waldmann, Jochen Schaible, Maja Stiawa, Thomas Becker, Karl Wegscheider, Bonnie Adema, Silke Wiegand-Grefe, Reinhold Kilian

**Affiliations:** 1 Department of Psychiatry and Psychotherapy at BKH Günzburg II, Ulm University, Lindenallee 2, 89312 Günzburg, Germany; 2 Institute for Medical Biometry and Epidemiology, University Medical Center Hamburg Eppendorf, Hamburg, Germany; 3 Department for Psychiatry and Psychotherapy, University Medical Center Hamburg Eppendorf, Hamburg, Germany; 4 Abteilung für Psychiatrie und Psychotherapie des Kindes- und Jugendalters, ZfP Südwürttemberg, Ravensburg, Germany; 5 Universitätsklinikum Leipzig, Klinik und Poliklinik für Psychiatrie und Psychotherapie, Leipzig, Germany

**Keywords:** Parent with mental illness with mental illness, Selective prevention, Children, Adolescents, Cost-utility, Health economic evaluation

## Abstract

**Background:**

Children of families with a parent with a mental illness have an increased risk of developing social and mental health problems resulting in decreased quality of life. Therefore, children and adolescents living in families with a parent with mental illness are regarded as a target group for preventive interventions. To date, only a few economic evaluation studies for interventions directed at preventing the intergenerational transmission of mental health problems exist. In this investigation we estimated the cost utility of an intervention for the support of children and adolescents with a parent having a mental illness from the perspective of the German health and social care system.

**Methods:**

We randomly assigned a total of 214 families with 337 children and adolescents to the intervention (INT) group (108/170) or the control (TAU) group (106/167). Families in the intervention group received on average eight intervention sessions (50–90 min) over 6 months. We estimated total cost of illness by means of the Children and Adolescent Mental Health Service Receipt Inventory (CAMHSRI) over 24 months. For the estimation of Quality-Adjusted Live Years (QALYs) we applied the KIDSCREEN-10. For estimating the incremental cost-utility of the intervention compared to treatment as usual we used the net-benefit approach.

**Results:**

We estimated the annual cost of illness amounting to € 3784.59 (SD € 8581.11) in the TAU group and € 3264.44 (SD € 9431.89) in the INT group. The annual cost difference between INT and TAU was € − 516.14 (SE 1124.95) which was not significant (*p* ≤ 0.05). We estimated the average QALY to be 0.759 (SD 0.073) in the TAU group and 0.763 (SD 0.072). The QALY difference between INT and TAU was 0.0037 (SE 0.0092) which was not significant (*p* ≤ 0.05). The incremental cost utility ratio (ICUR) indicated that the gain of one additional year in full health by means of the intervention was associated with the saving of € 139.49. However, the stochastic insecurity of the ICUR did not allow a unique decision about the cost-utility of the intervention.

**Conclusions:**

More information on the economic value of the intervention for families with a parent with mental illness in comparison to treatment as usual in Germany is needed.

*Trial registration*: ClinicalTrials.gov, identifier NCT02308462; German Clinical Trials Register: DRKS00006806.

**Supplementary Information:**

The online version contains supplementary material available at 10.1186/s13034-023-00693-w.

## Background

Worldwide, between 10 and 30% of children and adolescents are estimated to live with parents who have mental health problems [1–3]. Children of a parent with mental illness (COPMI) have an increased risk of developing mental health problems during childhood or adolescence themselves [1, 4–12]. This is not only true for severe mental disorders with a high heritability like psychosis or bipolar disorder but also for the whole spectrum of common mental disorders like depression, substance use, and anxiety [1]. The occurrence of a high prevalence of affected families and a high disease risk for the offspring makes COPMI a clearly defined target group for primary, secondary and tertiary mental disorder prevention [13, 14]. Although the process of the transgenerational transmission of mental disorders is still incompletely understood, most experts acknowledge the significant role of the rearing environment in this process beyond biological pathways [7, 15–17]. As one important result of recent research, effective treatment of maternal depression has been identified as a crucial factor in the prevention of the transmission of mental health problems [6, 7, 9]. Meanwhile, a broad spectrum of interventions for supporting affected families has been developed. Beyond strengthening the children’s coping skills and improving the communication between family members, interventions also target the mobilization of social support and professional networks [18–22]. In a systematic review and meta-analysis, Thanhäuser et al. [21] identified 53 studies including approximately 4500 individuals reporting the results of RCTs investigating the efficacy of interventions for the prevention of negative effects of parents’ mental disorders on mother-infant interactions and child psychopathology. The results of the meta-analyses indicate small effect sizes of approximately g = 0.33 on mother-infant interaction and even smaller but still significant effect sizes of approximately g = 0.13–0.17 on child psychopathology [21]. Lannes et al. [23] included data from 17 RCTs evaluating the effectiveness of preventive interventions for COPMI in a meta-analysis revealing a risk reduction of 47% in children to develop the same mental disorder as their parent. In addition, the authors identified a significant effect on internalizing symptoms during the 12 months after the intervention [23].

Compared to clinical efficacy, the economic efficiency of interventions for COPMI has only rarely been investigated thus far [24]. In a systematic review, Bee et al. identified one single study reporting the cost-effectiveness of an intervention to treat postnatal depression [24, 25]. Wansink et al. [26] investigated the cost-effectiveness of a preventive care-management program for families with a parent with mental illness with regard to the effects on parenting quality in comparison to treatment as usual (TAU). The intervention consisted of a five step preventive basic case management (PBCM) program with the main target on strengthening positive parenting and providing community and network support [26]. As outcome the authors used the improvement of parenting quality measured with the Home Observation for Measurement of the Environment Inventory (HOME) [27].

As a result of this study, the program was estimated to be cost-effective compared to TAU with a probability of 100% at a willingness to pay (WTP) of 2.500 €. That means that the program is cost-effective if the paying institution is willing to pay at least 2.500 € for the improvement of the outcome measure by one unit [26]. Since the authors used the family as the unit of analysis they could not apply a generalized outcome measure such as the QALY which is commonly used in health economic evaluations [28]. In contrast to the outcome measure applied by Wansink et al. generalized outcomes such as the QALY represent a combination of lifetime and quality of life. Health economic evaluation commonly report their results as cost per QALY which means the amount of money additional to the cost of TAU needed to gain on life year in full health by the intervention to be tested [29]. Therefore the results of most health economic evaluation studies can be directly compared independent of the type of intervention. However, generalized outcomes such as the QALY can only be estimated at the individual level and not on the group level [30] which means that even family focused interventions can only be evaluated at the individual level of each family member but not at the group level so far.

This makes it difficult to compare the results provided by Wansink et al. [26] with those of other studies.

Creswell et al. [31] investigated the cost-effectiveness of two interventions based on cognitive behavioural therapy (CBT). One intervention was dedicated to mother and child, while the second intervention was dedicated to preventing child anxiety disorders in children of mothers with anxiety disorders with the aim of improving combined quality adjusted life years for mothers and children [31]. The authors found that the combined CBT interventions were not more effective than child CBT alone. They concluded that the intervention was not cost-effective with regard to the WTP threshold between £ 20,000 and £ 30,000 recommended by the National Institute of Clinical Excellence (NICE) for the UK health care system [32, 33]. That means that the costs for the gain of 1 year of life in full health gained by the evaluated intervention increase £ 30,000 and that therefore the intervention would not be recommended by the NICE to become financed by the UK National Health Service [33].

Considering German-speaking countries, Pohl et al. [34] identified approximately 46 family-focused intervention programs for families with a parent having a mental illness in Germany, Austria and Switzerland. While 23 (54.8%) of these programs have been evaluated with regard to effectiveness, no health economic evaluation of any of these programs has been published thus far. This lack of evidence makes it difficult for decision makers to select programs that are suitable for implementation in routine health care.

In this article, we present the results of a cost-utility analysis for a family-based intervention for the primary, secondary and tertiary prevention of mental disorders in children and adolescents with parents having a mental illness from the perspective of the German health care system.

## Methods

In our description of the study methods and results we follow the consolidated health economic evaluation reporting standards (CHEERS) [35]  (see Additional file 1). The data for this health economic investigation were gathered as part of the study “Children of Parents with Mental Illness” CHIMPS [36].

### Trial design

We conducted a multi-centre randomized clinical trial (RCT) at six of the originally seven study sites which are located in Germany to evaluate the effectiveness and cost-effectiveness of a family-focused intervention for children with parents having a mental illness [36]. We excluded one study site located in Switzerland because of the differences in the health and social care systems between both countries. We collected data from all participating family members at baseline and at 6-, 12- and 18-month follow-ups. We recruited families at in- and outpatient departments of psychiatric hospitals for adults and for children and adolescents. Families were eligible for study participation if they had at least one child between ages 3 and 19 and if at least one parent was treated because of a common or severe mental illness during the last 5 years. Children or adolescents could be included with or without having a diagnosis of a mental illness. Exclusion criteria for parents and children or adolescents were acute symptoms such as suicidal tendencies, risk of self-harm and danger to others, acute psychotic symptoms, and other mental states with an indication for inpatient treatment. Eligible families were randomly assigned to the intervention or control group after the baseline assessment had been completed. Further details of the trial design, recruitment and randomization procedures are published in the study protocol [36].

### Intervention

Families in the intervention group received the intervention for children with a parent with mental illness (CHIMPS) [36, 37]. The aim of the intervention was the primary, secondary and tertiary prevention of mental illness in children or adolescents with at least one parent who had a common or severe mental illness. In case of children or adolescents without a diagnosis of a mental illness and without mental health problems at baseline the target of the intervention was the primary prevention of the onset of a mental disorder. In case of children or adolescents who already had mental health problems but did not fulfil the criteria of a diagnosis of a mental disorder at baseline the aim of the intervention was secondary prevention in the sense of detecting a potential mental disorder and giving advice for adequate treatment. In case of children or adolescents who already had a diagnosis of a mental disorder the aim of the study was tertiary prevention in the sense of improving the treatment. In addition, as a family focused program the intervention aims at improving psychological well-being in each family member.

CHIMPS is a manualized program [37] consisting on average of eight semi structured sessions (50–90 min) provided by a psychiatrist or psychotherapist over 6 months. Intervention providers were trained by the program developer. The program includes separate sessions with parents, each child and the entire family. The final number of sessions per family therefore depends on the number of participating family members. Further details of the CHIMPs intervention and the implementation of the program are provided in the study protocol [36].

### Control condition

Families assigned to the control condition received no additional services beyond the routine medical and psychiatric treatment and the psychosocial care provided by the German health, social care, child welfare, and the educational system. Routine health care is financed by mandatory or private health insurance and includes medical in- and outpatient hospital treatment, ambulant treatment by office-based family doctors and specialized physicians including psychiatrists, ambulant psychotherapy, other ambulant therapies and medication. In addition to health care financed by health insurance, support for families with special needs is provided by child and youth welfare services, which are tax-based financed by communities [38]. For children and adolescents with particular educational needs, several types of school-based services, such as school social workers or school psychologists, are available, which are tax-based financed by the communities or by the federal states [38].

### Perspective and scope of the health economic evaluation

In this article we present the health economic evaluation for the children and adolescents participating in the CHIMPS study. We will carry out a health economic evaluation for the participating parents in a separate analysis.

We conducted the health economic evaluation from the societal perspective. Therefore, we estimated total use and costs of health and psychosocial services including services provided by health care system, the child and youth welfare system and by the educational system. A detailed description of our cost assessment procedure is given in Waldmann et al. [38].

We conducted an incremental cost-utility analysis taking the child or adolescent as the unit of analysis from the perspective of the German health and social care welfare system. Therefore, only the data for children and adolescents from the six German study sites were included in this analysis. The analysis has a time frame of 24 months. For the incremental cost-utility analysis, we used an average 12-month time frame and two separate analyses for the first and second study years.

### Discounting

Due to the short time frame, we applied no discounting of costs and effects.

### Measures

#### Costs for health-care and psychosocial service use

We assessed the total use of health care and psychosocial services of the children and adolescents by means of the Children and Adolescent Mental Health Service Use Inventory (CAMHSRI) [39] adapted for the German health and social care system [38]. Due to the broad spectrum of needs related to mental health problems in children or adolescents we included the cost of health care but also the costs of psychosocial care provided by the child and youth welfare system and the costs of educational support provided by the educational system [38]. We estimated costs for service units reported to be used by the participating children or adolescents on the basis of literature and internet search and by personal consultation of service providers, health insurances and other payers [38].

#### Intervention costs

We estimated the intervention costs per child and per family. Although the aims of the intervention differed between children and adolescents and their parents we estimated the intervention costs as a whole because it was not possible to distinguish between children or adolescent and parent focused parts of the intervention.

On average, each family received eight intervention sessions, one initiating session with parents and children (60 min), two sessions with both parents (60 min), one session with each child (50 min) and three group sessions for the entire family (90 min). The intervention could be provided by psychiatrists or psychologists. Therefore, we calculated the costs for the intervention staff as € 102.57 per hour, representing the average hourly rate of a psychiatrist (€ 132.7) and a psychologist (€ 88.56). Given a total intervention time of 7 h, total intervention costs amounted to € 717.99 per family. Since each family had on average 1.6 children, we estimated costs per child by dividing the total family costs by 1.6 with the result of € 448.74 rounded to € 450.

### Outcomes

We measured the quality of life for children and adolescents by means of the KIDSCREEN-10 [40]. For the generation of quality adjusted life years we transformed the KIDSCREEN data into utility values by the algorithm provided by Chen et al. [41].

### Statistical analyses

We performed all statistical analyses on an intention-to-treat (ITT) basis using the last observation carried forward (LOCF) method for the imputation of missing data.

### Computation of average annual cost

We computed the average annual costs using the cost measures from baseline and the tree 6 months follow-up measures of 6 months cost as shown below:

$$Annual\,cost_{total} = \frac{{Cost_{t0} + Cost_{t1} + Cost_{t2} + Cost_{t3} }}{2}$$

where annual cost_total_ indicates the average annual cost of health and psychosocial care over 24 months and cost_t0_ to cost_t3_ indicate the 6 months cost measured retrospectively at baseline and the three follow-ups.

We collected information about service use retrospectively for the last 6 months before the time of assessment. Therefore the t0 cost assessment represents the cost over the 6 months before the baseline assessment. This makes the average annual costs computed by the formula above partly inert to being influenced by the intervention. The advantage of this approach is the availability of cost data in case of study drop-out after the baseline assessment. However, the disadvantage of this procedure is that the analysis becomes biased against the study hypothesis that total costs are influenced by the intervention. Therefore, we supplemented our overall analysis with two separate analyses for year one and year two. For this purpose we computed average annual cost for year one and for year two by means of the equations below:

$$\begin{aligned} Annual \,cost_{year 1} & = Cost_{t0} + Cost_{t1} \\ Annual \,cost_{year 2} & = Cost_{t2} + Cost_{t3} \\ \end{aligned}$$

where annual cost_year 1_ and annual cost_year 2_ indicate the average annual cost for the year one and year two and cost_t0_ to cost_t3_ indicate the 6 months cost measured retrospectively at baseline and the three follow-ups.

### Computation of QALYs

We computed average annual QALYs as the area under the curve [30]. For the overall analysis we computed the QALY by dividing the sum of the utility scores estimated from the KIDSCREEN-10 questionnaire by four as shown below:

$$QALY_{total} = \frac{{utility_{t0} + utility_{t1} + utility_{t2} + utility_{t3} }}{4}$$

where QALY_total_ indicates the average quality adjusted life years over the total study period. Utilityt0 to utilityt3 indicate the utility estimates based on the transformed KIDSCREEN-10 measures.

Division of the sum of the four utility scores by four is needed because we performed two utility assessments per year which means that each utility measure represents 0.5 QALYs [30].

Analogous to the cost assessment we estimated separate QALYs for each year by the formulas:

$$\begin{aligned} QALY_{year 1} & = \frac{{utility_{t0} + utility_{t1} }}{2} \\ QALY_{year 2} & = \frac{{utility_{t2} + utility_{t3} }}{2}. \\ \end{aligned}$$

### Statistical test of cost and QALY differences

For the assessment of the differences in cost and effects between study groups, we estimated linear regression models for costs and outcomes. We set the alpha error to *p* ≤ 0.05 taking into account that the children were clustered within families by including family identification as a cluster variable and by applying robust variance estimation [42]. In addition we took account for the skewed distribution of cost data by applying non-parametric bootstrapping with 2000 replications for estimating the 95% confidence intervals for the regression parameters [43] and we also estimated generalized linear models with gamma family distribution and logistic link functions [44] to confirm the inference of statistical differences between groups.

### Incremental cost-utility analyses

We computed e incremental cost-utility ratios (ICUR) as annual average over the total 24-month study period and separately for the year one and two. We interpreted the ICUR on the basis of its location at the cost effectiveness plane (CEP) [30].

For estimating the ICUR variance we carried out nonparametric bootstrapping with 10.000 replications [30]. We estimated the probability of cost-effectiveness depending on willingness to pay (WTP) thresholds between € 0 and € 125.000 by means of the cost-effectiveness acceptability curve (CEAC) [30]. In addition we estimatedthe probability of obtaining a net monetary benefit by means of net-benefit regression curves with 95% confidence intervals [30].

We conducted all analyses with Stata 16.1 using the programs provided by Henry A. Glick for estimating the ICUR variance, the acceptability curve and the net-monetary benefit regression [30].

## Results

**Fig. 1:**
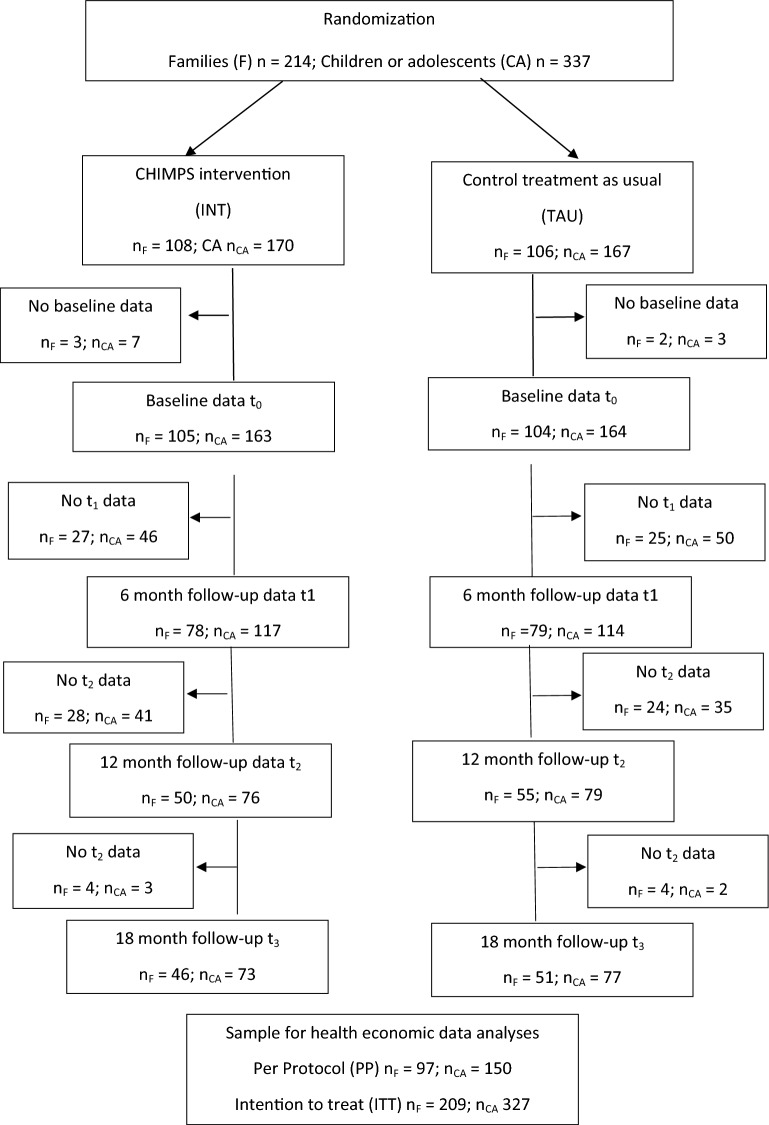
Study flow chart

From the 214 families with 337 children and adolescents randomly assigned to the intervention (INT) group (108/170) or the control (TAU) group (106/167), we included 327 children and adolescents (INT = 163; TAU = 164) from 209 families (INT = 105; TAU = 104) in the health economic evaluation (see Fig. 1). Due to missing cost or QALY data at baseline, we excluded 10 children or adolescents (INT = 6; TAU = 4) from 5 families (INT = 3; TAU = 2) from the health economic analyses. Due to missing data we performed LOCF imputation of cost data for 96 cases at t1, for 172 cases at t2 and for 170 cases at t3.

On average, the participants were 11.7 years old (sd = 4.4 years), and 169 (51.7%) were female. At baseline, 175 (53.5%) of the participating children or adolescents were diagnosed as having a mental illness. Statistical comparison of the aforementioned characteristics revealed no significant differences between study groups at baseline.

Table 1 (see Table 1) presents the 6-month costs of health and social care used by the children and adolescents broken down by study group. The largest share of costs in both groups were caused by the use of inpatient treatment with about 40% followed by school-based services with about 27% of the total costs. Costs for social and child or adolescent welfare services summed up to about 19%. Due to the large variance indicated by the standard deviation none of the cost differences are significant.

**Table 1:** Six-month costs of health and social care use at baseline

Cost category	Total sample	Control group	Intervention group	m Difference €	p Diff
	m €	m €	m €	Contr. versus Int.	Contr. versus Int.
	(SD)	(sd)	(sd)	(se)^a^	OLS^a^
					GLM^b^
Psychiatric and medical inpatient treatment	791.57 (3808.48)	1019.12 (4613.77)	562.63 (2768.50)	− 456.50 (423.79)	0.283
					0.268
Psychiatric and medical outpatient treatment	206.06 (579.93)	221.63 (600.72)	190.38 (559.65)	− 31.25 (71.72)	0.664
					0.665
Institutional child and adolescent welfare	224.45 (1862.13)	298.24 (2336.34)	150.21 (1214.35)	− 148.03 (203.29)	0.467
					0.431
Ambulant social or child and adolescent welfare	131.10 (529.56)	162.14 (669.46)	99.87 (333.82)	− 62.28 (75.64)	0.411
					0.348
Ambulant medication	19.89 (157.56)	15.60 (84.07)	24.22 (206.90)	8.62 (17.25)	0.618
					0.571
School based interventions	518.18 (2306.12)	551.79 (2451.68)	484.37 (2156.82)	− 67.41 (263.25)	0.798
					0.796
Total cost	1891.25 (5183.13)	2268.52 (5761.37)	1511.68 (4513.76)	− 756.84 (626.69)	0.229
					0.233

^a^Ordinary Least Square (OLS) regression with robust standard errors using family as cluster variable

^b^Generalized linear model (GLM) with gamma family and logistic link function and with family as cluster variable

**Fig. 2:**
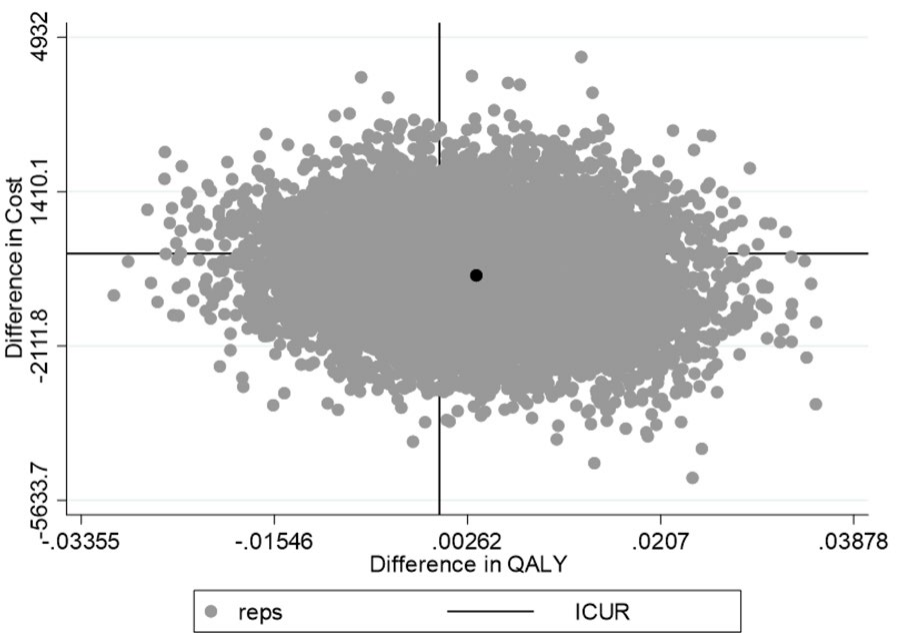
Variance of the incremental cost-utility ratio estimated by means of non-parametric bootstrapping with 10,000 replications. The x axis represents the difference in QALY between children in the TAU group and children in the INT group. The y axis represents the difference in annual health and social core costs between children in the TAU group and children in the INT group. The black dot represents the point estimate of the increment cost-utility ratio (ICUR). The grey dots represent the ICUR estimated by the 10.000 bootstrap resamples

Cost and effect differences are presented in Table 2. The average total annual cost over a period of 24 months was estimated to be € 3784.59 (SD € 8581.11) in the TAU group and € 3264.44 (SD € 9431.89) in the INT group, the annual cost difference between INT and TAU was € − 516.14 (SE 1124.95) which was not significant (*p* ≤ 0.05). The average QALY was estimated to be 0.759 (SD 0.073) in the TAU group and 0.763 (SD 0.072). The QALY difference between INT and TAU was 0.0037 (SE 0.0092) which was not significant (*p* ≤ 0.05). Based on cost and effect differences the incremental cost utility ratio (ICUR) was estimated as € − 139.49, indicating that the gain of one additional year in full health by means of the intervention was associated with the saving of € 139.49. However, the spread of the ICUR variance presented in Fig. 2 reveals an approximately uniform distribution over all four quadrants of the cost effectiveness analysis plane (CEP), indicating a high stochastic insecurity regarding the probability of the cost-effectiveness of the intervention.

**Table 2:** Costs, QALYs, and incremental cost utility ratios (ICUR)

	TAU	INT	∆ INT–TAU	p ∆^a^	ICUR
	mean	mean	(se)^a^		point estimate
	(sd)	(sd)			∆Cost/∆QALY
Annual average costs year 1 + 2	3784.59 (8581.11)	3264.44 (9431.89)	− 516.14 (1124.95)	0.647	€ − 139.49
Annual average QALY year 1 + 2	0.759 (0.073)	0.763 (0.072)	0.0037 (0.0092)	0.685	
Cost year 1	3723.22 (9241.54)	3387.0 (8816.41)	− 336.2 (1118.62)	0.764	€ − 105.06
QALY year 1	0.759 (0.068)	0.762 (0.066)	0.0032 (0.0087)	0.709	
Cost year 2	3845.95 (9819.97)	3149.90 (10,818.59)	− 696.05 (1267.75)	0. 7584	€ − 248.59
QALY year 2	0.760 (0.068)	0.763 (0.067)	0.0028 (0.0088)	0. 751	

*TAU* treatment as usual, *INT* intervention, *QALY* quality adjusted life year, *ICUR* incremental cost-utility ratio

^a^OLS regression with robust standard errors using nonparametric bootstrapping with 2000 replications

**Fig. 3:**
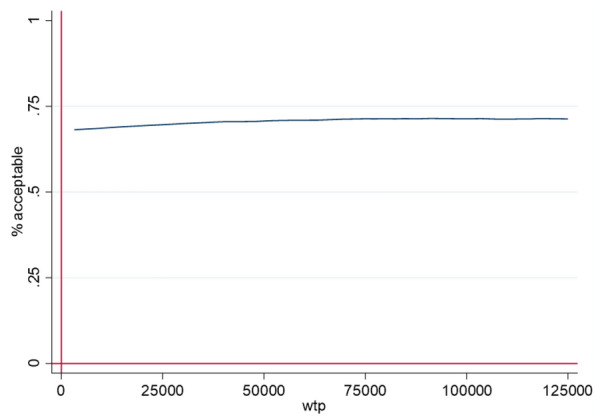
Cost-effectiveness acceptability curve (CEAC) for the cost-utility for the provision of the CHIMPs intervention for families with a parent with a mental illness in addition to treatment as usual in the German health and social care system. At the horizontal axis the CEAC shows potential values for MWTP in an increasing order, the vertical axis shows the percentages of the estimated ICUR values which are located below the MWTP curve. Similar as the statistical confidence interval the CEAC indicates at which MWTP a particular percentage of the estimated ICUR fall below the MWTP curve. A percentage of acceptance of 95% is regarded as equivalent to a one sided statistical significance of 2.5%. (wtp = willingness to pay in €)

This estimation is confirmed by the cost-effectiveness acceptability curve (CEAC) presented in Fig. 3, revealing that the probability that the intervention is cost-effective in comparison to TAU alone is below 75% for a WTP between 0 and € 125,000.

**Fig. 4:**
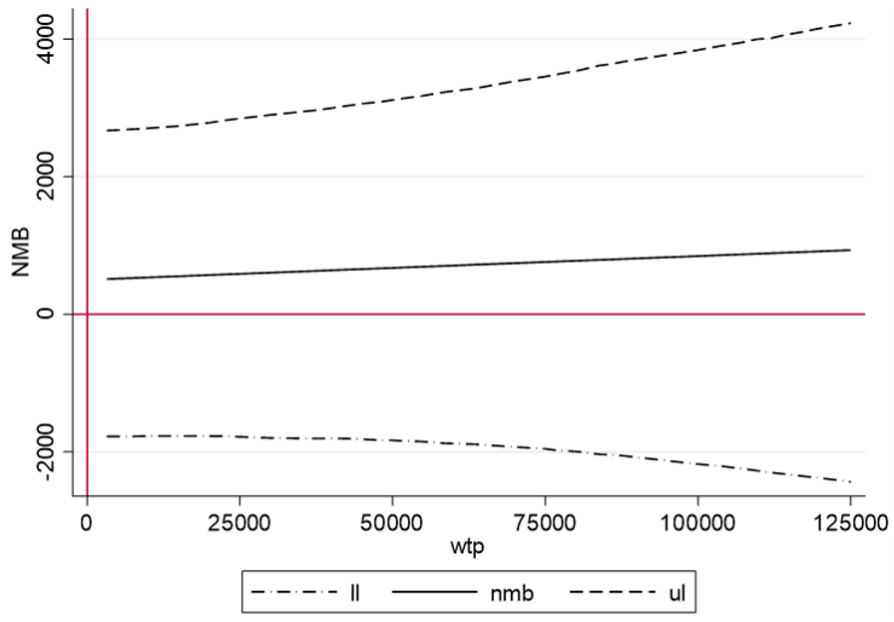
Net monetary benefit and 95% CI for the provision of the CHIMPs intervention for families with a parent with a mental illness in addition to treatment as usual in the German health and social care system. The net monetary benefit (NMB) regression curve represents the monetary gain which decision maker can expect from the implementation of the SH + intervention along a defined range of MWTP values between Euro zero and Euro 125,000. A positive NMB can be expected from the MWTP value where the lower limit of the 95% confidence interval of the NMB regression curve passes the x axis representing the MWTP (nmb = net monetary benefit in TRY; ll = lower limit of the 95% confidence interval; ul = upper limit of the 95% confidence interval; wtp = willingness to pay in €)

The net monetary benefit (NMB) curve in (see Fig. 4) indicates a positive net benefit over the WTP range from € 25,000 to 125,000. However, the limits of the 95% confidence interval reveal that the error probability of the net-benefit estimation largely increases to the limit of 5%.

## Discussion

This is the first health-economic evaluation of a family-focused intervention for children and adolescents with parents who have a mental disorder in Germany and one of the very few studies in this field worldwide [21, 24].

In contrast to the study of Wansink et al. [26], our study adds the use of a generalized outcome measure, which makes our results comparable to the majority of health economic evaluation studies across most areas of health and psychosocial care. In contrast to the study of Creswell et al. [31], the intervention evaluated in our investigation is not restricted to mothers and children with anxiety disorders but is applicable for parents with all types of mental disorders with children and adolescents across all states of social and mental health problems. In a pilot study including 67 families the intervention was found to be effective with regard to the improvement of children’s mental health and quality of life [37, 45]. However, in the current study we found no treatment effect on the quality of life of the participating children or adolescents. Perhaps, the broad range of applicability of the intervention might be one reason for the lack of effectiveness and cost-effectiveness revealed by our results. Analyses of service use and costs in our study sample revealed that families differed largely in the uptake of medical and psychosocial support [38]. This might be due to a broad variance regarding mental health problems but also due to a broad variance in unmet service needs [38]. Both sample characteristics may result in a weak average intervention effect and a large cost variance, as detected in our investigation. The intensity of the evaluated intervention could hardly be adjusted to the individual needs of the included families and children and adolescents. As a result, some families might have been offered more help than necessary, while others received too little support. Our cost analyses did not indicate significant changes in resource use, costs or cost utility over the period of investigation. The increased but still nonsignificant cost differences in the second study year result from the fact that intervention costs were only taken into account for the first study year.

Our finding of a lack of a significant treatment effect corresponds to the result reported by Creswell et al. [31], who also detected only a small and nonsignificant QALY difference of 0.02 resulting from a mother–child intervention. In contrast, Wansink et al. [26] reported intervention effects on parenting quality but not on children or adolescents’ quality of life. Therefore, it cannot be clearly concluded whether the interventions evaluated in our study or by Creswell et al. are less effective than those implemented by Wansink et al. because of the obtained effectiveness.

The authors of a recent systematic review and meta-analysis of 33 RCTs with 3020 cases evaluating interventions for children and adolescents with parents with mental illness [21] identified an average overall effect size for child psychopathology of 0.13, which is rather small regarding usual classifications [46, 47]. Unfortunately, none of the studies included in the review by Thanhäuser et al. reported quality of life or QALY outcomes [21].

This lack of studies reporting quality of life or QALYs as outcomes in investigations on the mental health of children and adolescents in general [48] and in studies for the evaluation of interventions for supporting families with parents having a mental illness in particular makes it difficult to derive unambiguous conclusions from the results of our study thus far. On the one hand, our results suggest that the effects of the intervention on the gain of QALYs are too weak to cause any significant treatment effect. On the other hand, the measure of QALYs might not be sensitive enough to reflect small clinical changes [48].

### Strengths and limitations

This is the first study evaluating the cost utility of an intervention for the support of families with a parent with mental illness in Germany in an RCT study design. The strengths of the study are the duration of 18 months, a comprehensive assessment of costs including health and psychosocial care, child welfare and school-based services, and the application of a generalized outcome measure.

Limitations result mainly from the high sample attrition rate of 177 (54%) participants from baseline to t3. Limitations also result from the cost assessment on the basis of self-reports, which makes cost data susceptible to memory bias.

Limitations of the study also result from the inclusion of German study sites only. This results in the restriction of the generalizability of our results to the context of the German health and social care system.

Limitations result also from restricting our analysis to the perspective of the children and adolescents instead of that of the whole family.

## Conclusions

Study results allow no clear conclusion about the cost effectiveness of the intervention in comparison to treatment as usual. Improving the state of knowledge about the cost-effectiveness of the target intervention would be of significant economic value for the German health and social care system.

### Supplementary Information

**Additional file 1.** Consolidated Health EconomicEvaluation Reporting Standards (CHEERS) checklist.

## Data Availability

Data include sensitive mental health information about minor participants. In the written consent form the study participants were assured that data would not be shared with third parties. Therefor the complete data cannot be openly shared.
